# *N*,*N*′-Bis[(*E*)-4-nitro­benzyl­idene]-4,4′-oxydianiline

**DOI:** 10.1107/S1600536809033844

**Published:** 2009-08-29

**Authors:** Hee K. Lee, Soon W. Lee

**Affiliations:** a Department of Chemistry (BK21), Sungkyunkwan University, Natural Science Campus, Suwon 440-746, Republic of Korea

## Abstract

The title compound, C_26_H_18_N_4_O_5_, can be regarded as an extended ether with two terminal nitro groups. The two aryl rings bonded to the central O atom form a dihedral angle of 75.72 (6)°, and the terminal nitro groups are slightly twisted [by 6.4 (2) and 3.3 (3)°] from the benzene rings to which they are attached. The crystal packing exhibits weak inter­molecular C—H⋯O hydrogen bonds and π–π inter­actions [centroid–centroid distances = 3.794 (3) Å].

## Related literature

For applications of coordination polymers, see: Barnett & Champness (2003 ▶); Batten *et al.* (2009 ▶); Perry *et al.* (2009 ▶). For bis­(pyridine)-, bis­(furan)-, bis­(thio­phene)-, and (pyridine–amine)-type linking ligands as well as compounds that are structurally close to the title compound, see Yun *et al.* (2009 ▶) and references therein.
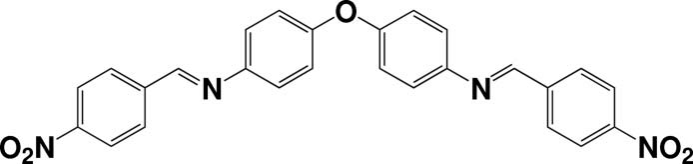


## Experimental

### 

#### Crystal data

C_26_H_18_N_4_O_5_
*M* *_r_* = 466.44
Triclinic, 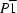

*a* = 8.3322 (11) Å
*b* = 9.0716 (16) Å
*c* = 17.107 (2) Å
α = 74.714 (9)°
β = 78.885 (10)°
γ = 64.643 (10)°
*V* = 1122.4 (3) Å^3^
*Z* = 2
Mo *K*α radiation
μ = 0.10 mm^−1^
*T* = 293 K
0.52 × 0.32 × 0.26 mm

#### Data collection

Siemens P4 diffractometer
Absorption correction: none
4200 measured reflections
3909 independent reflections
2972 reflections with *I* > 2σ(*I*)
*R*_int_ = 0.015
3 standard reflections every 97 reflections intensity decay: none

#### Refinement

*R*[*F* ^2^ > 2σ(*F* ^2^)] = 0.041
*wR*(*F* ^2^) = 0.114
*S* = 1.02
3909 reflections
389 parameters
All H-atom parameters refined
Δρ_max_ = 0.14 e Å^−3^
Δρ_min_ = −0.19 e Å^−3^

### 

Data collection: *XSCANS* (Siemens, 1995 ▶); cell refinement: *XSCANS*; data reduction: *XSCANS*; program(s) used to solve structure: *SHELXTL* (Sheldrick, 2008 ▶); program(s) used to refine structure: *SHELXTL*; molecular graphics: *SHELXTL*; software used to prepare material for publication: *SHELXTL*.

## Supplementary Material

Crystal structure: contains datablocks global, I. DOI: 10.1107/S1600536809033844/cv2603sup1.cif

Structure factors: contains datablocks I. DOI: 10.1107/S1600536809033844/cv2603Isup2.hkl

Additional supplementary materials:  crystallographic information; 3D view; checkCIF report

## Figures and Tables

**Table 1:** Hydrogen-bond geometry (Å, °)

*D*—H⋯*A*	*D*—H	H⋯*A*	*D*⋯*A*	*D*—H⋯*A*
C19—H19⋯O1^i^	0.956 (19)	2.57 (2)	3.375 (2)	142.4 (14)
C20—H20⋯O5^ii^	1.016 (19)	2.48 (2)	3.323 (2)	139.7 (14)

Symmetry codes: (i) 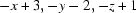; (ii) 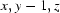.

**Fig. 1.**
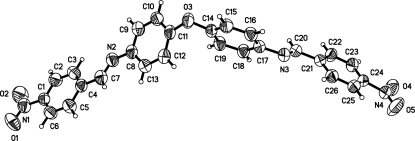
Molecular structure of the title compound showing 50% probability displacement ellipsoids.

## supplementary crystallographic information

### Comment

Coordination polymers gain continuous attention due to their desirable zeolite-like properties applicable to catalysis, nonlinear optical activity, spin crossover, luminescence, long-range magnetism, adsorption-desorption, and gas storage (Barnett & Champness, 2003; Batten *et al.*, 2009; Perry IV *et al.*, 2009). Carefule choice of relevant linking ligands is one of the key factors for the successful preparation of such polymers. We have continually reported long bis(pyridine)-, bis(furan)-, bis(thiophene)-, and (pyridine–amine)-type linking ligands and their coordination polymers (Yun *et al.*, 2009). To an extension of our ongoing study of novel linking ligands and their coordination polymers, we prepared a long, potential linking ligand containing two terminal nitro groups.

The molecular structure of the title compound with the atom-numbering scheme is shown in Fig. 1. The overall structure can be regarded as a long ether possessing two terminal nitro (NO_2_) groups. Two bis(pyridine)-type linking ligands containing an intervening oxydianilne fragment, which are structurally close to the title compound, were recently reported by our research group: [(3-py)—CHN—C_6_H_4_—O—C_6_H_4_—NCH—(3-py)] and [(4-py)—CHN—C_6_H_4_—O—C_6_H_4_—NCH—(4-py)] (Yun *et al.*, 2009), which, however, were not structurally characterized. In the title compound, the dihedral angle between two aryl rings (C8–13 and C14–C19) bonded to the central oxygen (O13) is 75.72 (6)°. Terminal nitro groups are not coplanar with the phenyl rings to which they are attached, with the dihedral angle 6.4 (2) (N1, O1, O2) or 3.3 (3)° (N2, O4, O5). These bonding parameters might indicate the flexibility of the title compound. The crystal packing exhibits weak intermolecular C—H···O hydrogen bonds (Table 1) and π–π interactions proved by centroid-to-centroid distances of 3.794 (3) Å.

### Experimental

4-Nitrobenzaldehyde (1.12 g, 7.41 mmol) and 4,4-oxydianiline (0.67 g, 3.31 mmol) were dissolved in methanol (80 ml), to which formic acid (0.15 ml) was added. The resulting mixture was stirred at room temperature for 1 h. Dichloromethane (50 ml) was then added to the mixture, which was further stirred for 24 h. The resulting solution was filtered to give a yellow solid, which was washed with hexane (20 ml × 2). X-ray quality crystals were obtained from dichloromethane/hexane. Yield: 92%. mp: 457–459 K. IR (KBr, cm^-1^): 3427, 3099, 2846, 2441, 1595, 1517, 1490, 1340, 1240, 1104, 850.

### Refinement

All H atoms were located on a Fourier difference map and refined isotropically.

### Crystal data

**Table:**

C_26_H_18_N_4_O_5_	*Z* = 2
*M**_r_* = 466.44	*F*(000) = 484
Triclinic, *P*1	*D*_x_ = 1.380 Mg m^−^^3^
Hall symbol: -P 1	Mo *K*α radiation, λ = 0.71073 Å
*a* = 8.3322 (11) Å	Cell parameters from 23 reflections
*b* = 9.0716 (16) Å	θ = 5.2–12.5°
*c* = 17.107 (2) Å	µ = 0.10 mm^−^^1^
α = 74.714 (9)°	*T* = 293 K
β = 78.885 (10)°	Block, yellow
γ = 64.643 (10)°	0.52 × 0.32 × 0.26 mm
*V* = 1122.4 (3) Å^3^	

### Data collection

**Table:**

Siemens P4 diffractometer	*R*_int_ = 0.015
Radiation source: sealed tube	θ_max_ = 25.0°, θ_min_ = 2.5°
graphite	*h* = 0→9
ω scans	*k* = −9→10
4200 measured reflections	*l* = −19→20
3909 independent reflections	3 standard reflections every 97 reflections
2972 reflections with *I* > 2σ(*I*)	intensity decay: none

### Refinement

**Table:**

Refinement on *F*^2^	Secondary atom site location: difference Fourier map
Least-squares matrix: full	Hydrogen site location: inferred from neighbouring sites
*R*[*F*^2^ > 2σ(*F*^2^)] = 0.041	All H-atom parameters refined
*wR*(*F*^2^) = 0.114	*w* = 1/[σ^2^(*F*_o_^2^) + (0.0503*P*)^2^ + 0.1958*P*] where *P* = (*F*_o_^2^ + 2*F*_c_^2^)/3
*S* = 1.02	(Δ/σ)_max_ < 0.001
3909 reflections	Δρ_max_ = 0.14 e Å^−^^3^
389 parameters	Δρ_min_ = −0.19 e Å^−^^3^
0 restraints	Extinction correction: *SHELXTL* (Sheldrick, 2008), Fc^*^=kFc[1+0.001xFc^2^λ^3^/sin(2θ)]^-1/4^
Primary atom site location: structure-invariant direct methods	Extinction coefficient: 0.0142 (18)

### Special details

**Table:**

Geometry. All e.s.d.'s (except the e.s.d. in the dihedral angle between two l.s. planes) are estimated using the full covariance matrix. The cell e.s.d.'s are taken into account individually in the estimation of e.s.d.'s in distances, angles and torsion angles; correlations between e.s.d.'s in cell parameters are only used when they are defined by crystal symmetry. An approximate (isotropic) treatment of cell e.s.d.'s is used for estimating e.s.d.'s involving l.s. planes.
Refinement. Refinement of *F*^2^ against ALL reflections. The weighted *R*-factor *wR* and goodness of fit *S* are based on *F*^2^, conventional *R*-factors *R* are based on *F*, with *F* set to zero for negative *F*^2^. The threshold expression of *F*^2^ > σ(*F*^2^) is used only for calculating *R*-factors(gt) *etc*. and is not relevant to the choice of reflections for refinement. *R*-factors based on *F*^2^ are statistically about twice as large as those based on *F*, and *R*- factors based on ALL data will be even larger.

### Fractional atomic coordinates and isotropic or equivalent isotropic displacement parameters (Å^2^)

**Table:**

	*x*	*y*	*z*	*U*_iso_*/*U*_eq_	
O1	1.7461 (2)	−1.5786 (2)	0.74989 (10)	0.1006 (6)	
O2	1.8293 (3)	−1.6837 (2)	0.64341 (12)	0.1072 (6)	
O3	0.66294 (17)	−0.41925 (15)	0.27173 (8)	0.0675 (4)	
O4	0.8910 (2)	0.87019 (18)	−0.12942 (11)	0.0838 (5)	
O5	0.7292 (3)	0.9709 (2)	−0.02700 (11)	0.1184 (7)	
N1	1.7396 (2)	−1.5702 (2)	0.67832 (12)	0.0737 (5)	
N2	1.2107 (2)	−0.96099 (19)	0.43282 (10)	0.0631 (4)	
N3	0.6330 (2)	0.22173 (17)	0.13724 (9)	0.0556 (4)	
N4	0.7983 (2)	0.86027 (19)	−0.06546 (11)	0.0667 (4)	
C1	1.6151 (2)	−1.4143 (2)	0.63208 (11)	0.0561 (4)	
C2	1.5930 (3)	−1.4084 (3)	0.55334 (13)	0.0697 (5)	
C3	1.4715 (3)	−1.2638 (3)	0.51122 (13)	0.0692 (5)	
C4	1.3747 (2)	−1.1275 (2)	0.54727 (11)	0.0567 (4)	
C5	1.4043 (3)	−1.1372 (3)	0.62583 (12)	0.0666 (5)	
C6	1.5244 (3)	−1.2814 (3)	0.66859 (12)	0.0646 (5)	
C7	1.2366 (3)	−0.9765 (3)	0.50481 (13)	0.0640 (5)	
C8	1.0753 (2)	−0.8152 (2)	0.39338 (11)	0.0557 (4)	
C9	1.0170 (3)	−0.8290 (2)	0.32617 (11)	0.0614 (5)	
C10	0.8827 (3)	−0.6950 (2)	0.28467 (12)	0.0603 (5)	
C11	0.8091 (2)	−0.5460 (2)	0.30985 (11)	0.0554 (4)	
C12	0.8703 (3)	−0.5267 (2)	0.37428 (12)	0.0640 (5)	
C13	1.0025 (3)	−0.6607 (2)	0.41617 (13)	0.0650 (5)	
C14	0.6656 (2)	−0.2624 (2)	0.24132 (10)	0.0527 (4)	
C15	0.5020 (2)	−0.1291 (2)	0.23764 (11)	0.0570 (4)	
C16	0.4942 (2)	0.0295 (2)	0.20338 (11)	0.0560 (4)	
C17	0.6481 (2)	0.0556 (2)	0.17089 (9)	0.0504 (4)	
C18	0.8120 (2)	−0.0792 (2)	0.17666 (11)	0.0565 (4)	
C19	0.8211 (2)	−0.2388 (2)	0.21218 (11)	0.0569 (4)	
C20	0.7171 (2)	0.2500 (2)	0.06917 (11)	0.0534 (4)	
C21	0.7241 (2)	0.4137 (2)	0.03302 (10)	0.0495 (4)	
C22	0.8183 (3)	0.4350 (2)	−0.04281 (11)	0.0580 (5)	
C23	0.8408 (3)	0.5821 (2)	−0.07591 (12)	0.0588 (5)	
C24	0.7657 (2)	0.70731 (19)	−0.03286 (11)	0.0527 (4)	
C25	0.6657 (3)	0.6929 (2)	0.04074 (11)	0.0606 (5)	
C26	0.6447 (3)	0.5455 (2)	0.07374 (11)	0.0567 (4)	
H2	1.657 (3)	−1.501 (3)	0.5313 (12)	0.079 (6)*	
H3	1.454 (3)	−1.258 (3)	0.4561 (14)	0.089 (7)*	
H5	1.332 (3)	−1.034 (3)	0.6515 (13)	0.089 (7)*	
H6	1.542 (3)	−1.293 (2)	0.7242 (13)	0.077 (6)*	
H7	1.167 (3)	−0.892 (3)	0.5362 (14)	0.099 (8)*	
H9	1.073 (3)	−0.939 (2)	0.3098 (11)	0.072 (6)*	
H10	0.839 (3)	−0.707 (2)	0.2398 (12)	0.077 (6)*	
H12	0.822 (3)	−0.421 (3)	0.3907 (12)	0.075 (6)*	
H13	1.044 (2)	−0.650 (2)	0.4595 (12)	0.063 (5)*	
H15	0.397 (3)	−0.151 (2)	0.2596 (11)	0.071 (6)*	
H16	0.381 (3)	0.125 (2)	0.2019 (11)	0.071 (6)*	
H18	0.923 (3)	−0.061 (2)	0.1556 (11)	0.063 (5)*	
H19	0.934 (3)	−0.331 (2)	0.2162 (11)	0.064 (5)*	
H20	0.784 (3)	0.161 (2)	0.0355 (11)	0.066 (5)*	
H22	0.876 (3)	0.342 (2)	−0.0724 (11)	0.071 (6)*	
H23	0.904 (3)	0.597 (2)	−0.1244 (12)	0.075 (6)*	
H25	0.616 (3)	0.777 (2)	0.0689 (12)	0.071 (6)*	
H26	0.577 (3)	0.528 (2)	0.1242 (12)	0.069 (6)*	

### Atomic displacement parameters (Å^2^)

**Table:**

	*U*^11^	*U*^22^	*U*^33^	*U*^12^	*U*^13^	*U*^23^
O1	0.0832 (11)	0.1146 (13)	0.0768 (11)	−0.0193 (10)	−0.0332 (9)	0.0079 (9)
O2	0.1096 (14)	0.0678 (10)	0.1184 (14)	−0.0056 (10)	−0.0316 (11)	−0.0138 (10)
O3	0.0578 (8)	0.0524 (7)	0.0882 (9)	−0.0272 (6)	−0.0217 (7)	0.0123 (6)
O4	0.0881 (11)	0.0700 (9)	0.0968 (11)	−0.0467 (8)	−0.0059 (9)	0.0018 (8)
O5	0.200 (2)	0.0732 (10)	0.1070 (13)	−0.0782 (13)	0.0012 (13)	−0.0290 (10)
N1	0.0614 (11)	0.0742 (12)	0.0796 (13)	−0.0268 (9)	−0.0185 (9)	0.0020 (10)
N2	0.0586 (9)	0.0617 (9)	0.0639 (10)	−0.0236 (8)	−0.0106 (8)	−0.0018 (7)
N3	0.0617 (9)	0.0480 (8)	0.0558 (9)	−0.0231 (7)	−0.0096 (7)	−0.0031 (6)
N4	0.0812 (12)	0.0512 (9)	0.0746 (11)	−0.0319 (9)	−0.0258 (9)	−0.0009 (8)
C1	0.0512 (10)	0.0583 (10)	0.0584 (11)	−0.0253 (9)	−0.0112 (8)	−0.0007 (8)
C2	0.0724 (13)	0.0627 (12)	0.0707 (13)	−0.0205 (11)	−0.0128 (10)	−0.0147 (10)
C3	0.0763 (13)	0.0713 (13)	0.0579 (12)	−0.0268 (11)	−0.0163 (10)	−0.0073 (10)
C4	0.0542 (10)	0.0590 (10)	0.0568 (10)	−0.0278 (9)	−0.0060 (8)	−0.0018 (8)
C5	0.0674 (12)	0.0655 (12)	0.0615 (12)	−0.0217 (10)	−0.0082 (10)	−0.0108 (9)
C6	0.0667 (12)	0.0721 (13)	0.0549 (11)	−0.0286 (10)	−0.0128 (9)	−0.0061 (9)
C7	0.0611 (12)	0.0633 (12)	0.0619 (12)	−0.0249 (10)	−0.0042 (9)	−0.0045 (10)
C8	0.0493 (10)	0.0543 (10)	0.0570 (10)	−0.0224 (8)	−0.0051 (8)	0.0020 (8)
C9	0.0598 (11)	0.0569 (11)	0.0606 (11)	−0.0197 (9)	−0.0048 (9)	−0.0074 (9)
C10	0.0596 (11)	0.0620 (11)	0.0575 (11)	−0.0259 (9)	−0.0091 (9)	−0.0037 (9)
C11	0.0492 (10)	0.0511 (10)	0.0598 (10)	−0.0242 (8)	−0.0069 (8)	0.0071 (8)
C12	0.0705 (13)	0.0498 (10)	0.0674 (12)	−0.0228 (9)	−0.0100 (10)	−0.0047 (9)
C13	0.0733 (13)	0.0614 (12)	0.0618 (12)	−0.0294 (10)	−0.0179 (10)	−0.0022 (9)
C14	0.0561 (10)	0.0483 (9)	0.0528 (10)	−0.0247 (8)	−0.0109 (8)	0.0019 (7)
C15	0.0475 (10)	0.0582 (11)	0.0636 (11)	−0.0237 (9)	−0.0086 (8)	−0.0025 (8)
C16	0.0490 (10)	0.0518 (10)	0.0607 (11)	−0.0156 (8)	−0.0112 (8)	−0.0043 (8)
C17	0.0575 (10)	0.0481 (9)	0.0454 (9)	−0.0227 (8)	−0.0089 (8)	−0.0033 (7)
C18	0.0519 (10)	0.0543 (10)	0.0615 (11)	−0.0248 (9)	−0.0053 (8)	−0.0024 (8)
C19	0.0484 (10)	0.0509 (10)	0.0634 (11)	−0.0176 (8)	−0.0083 (8)	−0.0003 (8)
C20	0.0603 (11)	0.0473 (9)	0.0549 (10)	−0.0230 (8)	−0.0103 (8)	−0.0080 (8)
C21	0.0536 (10)	0.0456 (9)	0.0499 (9)	−0.0201 (8)	−0.0122 (7)	−0.0048 (7)
C22	0.0698 (12)	0.0462 (10)	0.0567 (11)	−0.0224 (9)	−0.0026 (9)	−0.0119 (8)
C23	0.0635 (11)	0.0524 (10)	0.0573 (11)	−0.0245 (9)	−0.0026 (9)	−0.0057 (8)
C24	0.0592 (10)	0.0431 (9)	0.0589 (10)	−0.0221 (8)	−0.0202 (8)	−0.0018 (7)
C25	0.0757 (13)	0.0463 (10)	0.0595 (11)	−0.0190 (9)	−0.0140 (10)	−0.0137 (8)
C26	0.0651 (11)	0.0524 (10)	0.0507 (10)	−0.0219 (9)	−0.0066 (9)	−0.0091 (8)

### Geometric parameters (Å, °)

**Table:**

O1—N1	1.217 (2)	C10—C11	1.373 (3)
O2—N1	1.211 (2)	C10—H10	0.96 (2)
O3—C14	1.388 (2)	C11—C12	1.379 (3)
O3—C11	1.394 (2)	C12—C13	1.379 (3)
O4—N4	1.218 (2)	C12—H12	0.97 (2)
O5—N4	1.216 (2)	C13—H13	0.920 (19)
N1—C1	1.474 (2)	C14—C19	1.380 (2)
N2—C7	1.252 (3)	C14—C15	1.381 (2)
N2—C8	1.422 (2)	C15—C16	1.381 (2)
N3—C20	1.262 (2)	C15—H15	0.97 (2)
N3—C17	1.424 (2)	C16—C17	1.387 (2)
N4—C24	1.472 (2)	C16—H16	0.97 (2)
C1—C6	1.360 (3)	C17—C18	1.389 (2)
C1—C2	1.378 (3)	C18—C19	1.389 (2)
C2—C3	1.382 (3)	C18—H18	0.990 (19)
C2—H2	0.92 (2)	C19—H19	0.956 (19)
C3—C4	1.384 (3)	C20—C21	1.472 (2)
C3—H3	0.97 (2)	C20—H20	1.016 (19)
C4—C5	1.386 (3)	C21—C26	1.392 (2)
C4—C7	1.475 (3)	C21—C22	1.393 (2)
C5—C6	1.380 (3)	C22—C23	1.383 (3)
C5—H5	1.03 (2)	C22—H22	0.99 (2)
C6—H6	0.96 (2)	C23—C24	1.371 (3)
C7—H7	0.96 (2)	C23—H23	0.90 (2)
C8—C9	1.385 (3)	C24—C25	1.378 (3)
C8—C13	1.397 (3)	C25—C26	1.378 (3)
C9—C10	1.386 (3)	C25—H25	0.92 (2)
C9—H9	0.99 (2)	C26—H26	0.953 (19)
			
C14—O3—C11	118.85 (13)	C13—C12—H12	119.3 (12)
O2—N1—O1	123.39 (19)	C12—C13—C8	120.5 (2)
O2—N1—C1	118.48 (19)	C12—C13—H13	120.5 (12)
O1—N1—C1	118.1 (2)	C8—C13—H13	119.0 (12)
C7—N2—C8	120.80 (18)	C19—C14—C15	120.83 (16)
C20—N3—C17	118.44 (15)	C19—C14—O3	122.74 (16)
O5—N4—O4	123.56 (17)	C15—C14—O3	116.35 (15)
O5—N4—C24	117.74 (18)	C14—C15—C16	119.40 (17)
O4—N4—C24	118.69 (16)	C14—C15—H15	118.0 (11)
C6—C1—C2	122.07 (18)	C16—C15—H15	122.6 (11)
C6—C1—N1	119.08 (18)	C15—C16—C17	120.79 (17)
C2—C1—N1	118.85 (19)	C15—C16—H16	120.6 (12)
C1—C2—C3	118.6 (2)	C17—C16—H16	118.6 (12)
C1—C2—H2	119.0 (13)	C16—C17—C18	119.11 (15)
C3—C2—H2	122.4 (13)	C16—C17—N3	118.57 (15)
C2—C3—C4	120.64 (19)	C18—C17—N3	122.20 (16)
C2—C3—H3	119.7 (13)	C17—C18—C19	120.35 (17)
C4—C3—H3	119.6 (13)	C17—C18—H18	119.4 (10)
C3—C4—C5	118.88 (18)	C19—C18—H18	120.2 (10)
C3—C4—C7	121.08 (18)	C14—C19—C18	119.44 (17)
C5—C4—C7	119.99 (19)	C14—C19—H19	120.4 (11)
C6—C5—C4	120.9 (2)	C18—C19—H19	120.2 (11)
C6—C5—H5	121.3 (12)	N3—C20—C21	122.54 (17)
C4—C5—H5	117.8 (12)	N3—C20—H20	122.0 (10)
C1—C6—C5	118.84 (19)	C21—C20—H20	115.4 (10)
C1—C6—H6	118.9 (12)	C26—C21—C22	119.25 (16)
C5—C6—H6	122.2 (12)	C26—C21—C20	121.80 (16)
N2—C7—C4	121.7 (2)	C22—C21—C20	118.91 (16)
N2—C7—H7	122.5 (14)	C23—C22—C21	120.78 (17)
C4—C7—H7	115.8 (14)	C23—C22—H22	119.2 (11)
C9—C8—C13	118.62 (17)	C21—C22—H22	120.0 (11)
C9—C8—N2	116.51 (17)	C24—C23—C22	118.32 (18)
C13—C8—N2	124.84 (17)	C24—C23—H23	119.8 (13)
C8—C9—C10	120.91 (19)	C22—C23—H23	121.9 (13)
C8—C9—H9	117.4 (11)	C23—C24—C25	122.38 (16)
C10—C9—H9	121.7 (11)	C23—C24—N4	118.52 (17)
C11—C10—C9	119.33 (19)	C25—C24—N4	119.07 (16)
C11—C10—H10	120.1 (12)	C24—C25—C26	119.05 (17)
C9—C10—H10	120.6 (12)	C24—C25—H25	121.6 (12)
C10—C11—C12	120.95 (17)	C26—C25—H25	119.3 (12)
C10—C11—O3	117.62 (17)	C25—C26—C21	120.13 (18)
C12—C11—O3	121.29 (17)	C25—C26—H26	123.1 (12)
C11—C12—C13	119.60 (19)	C21—C26—H26	116.8 (12)
C11—C12—H12	121.1 (12)		

### Hydrogen-bond geometry (Å, °)

**Table:**

*D*—H···*A*	*D*—H	H···*A*	*D*···*A*	*D*—H···*A*
C19—H19···O1^i^	0.956 (19)	2.57 (2)	3.375 (2)	142.4 (14)
C20—H20···O5^ii^	1.016 (19)	2.48 (2)	3.323 (2)	139.7 (14)

Symmetry codes: (i) −*x*+3, −*y*−2, −*z*+1; (ii) *x*, *y*−1, *z*.
